# Prediction using step-wise L1, L2 regularization and feature selection for small data sets with large number of features

**DOI:** 10.1186/1471-2105-12-412

**Published:** 2011-10-25

**Authors:** Ozgur Demir-Kavuk, Mayumi Kamada, Tatsuya Akutsu, Ernst-Walter Knapp

**Affiliations:** 1 Institute of Chemistry and Biochemistry, Freie Universität Berlin, Fabeckstrasse 36A, 14195 Berlin, Germany; 2 Bioinformatics Center, Institute for Chemical Research, Kyoto University, Uji, Kyoto 611-0011, Japan

## Abstract

**Background:**

Machine learning methods are nowadays used for many biological prediction problems involving drugs, ligands or polypeptide segments of a protein. In order to build a prediction model a so called training data set of molecules with measured target properties is needed. For many such problems the size of the training data set is limited as measurements have to be performed in a wet lab. Furthermore, the considered problems are often complex, such that it is not clear which molecular descriptors (features) may be suitable to establish a strong correlation with the target property. In many applications all available descriptors are used. This can lead to difficult machine learning problems, when thousands of descriptors are considered and only few (e.g. below hundred) molecules are available for training.

**Results:**

The CoEPrA contest provides four data sets, which are typical for biological regression problems (few molecules in the training data set and thousands of descriptors). We applied the same two-step training procedure for all four regression tasks. In the first stage, we used optimized L1 regularization to select the most relevant features. Thus, the initial set of more than 6,000 features was reduced to about 50. In the second stage, we used only the selected features from the preceding stage applying a milder L2 regularization, which generally yielded further improvement of prediction performance. Our linear model employed a soft loss function which minimizes the influence of outliers.

**Conclusions:**

The proposed two-step method showed good results on all four CoEPrA regression tasks. Thus, it may be useful for many other biological prediction problems where for training only a small number of molecules are available, which are described by thousands of descriptors.

## Background

Nowadays, empirical methods of machine learning are widely used in life sciences and related sciences such as chemistry, biochemistry, pharmacy, and medicinal diagnostics. They can be used to predict the value of a target property in focus such as the competence of a molecular compound to fulfill a specific function. For this purpose, regression methods correlate specific molecular properties (features or descriptors) of molecular compounds with the desired target property. In the simplest case, the regression uses an objective function whose number of parameters is as large as the number of considered features. Usually the objective function contains also a so-called regularization term. It penalizes model details of unnecessary complexity, focuses on the most relevant features, and thus avoids over-fitting of the data used for training (parameter optimization) [1]. The most commonly used regularization methods are L1 regularization, also known as Lasso [2] and L2 regularization also known as ridge regression [3]. As penalty term, the L1 regularization adds the sum of the absolute values of the model parameters to the objective function whereas the L2 regularization adds the sum of the squares of them. Due to its inherent linear dependence on the model parameters, regularization with L1 disables irrelevant features leading to sparse sets of features. Thus, L1 regularization combines efficient feature selection and model generation into one single optimization step.

In recent years, considerable advancements were made in high throughput techniques to generate for a large number of relevant molecular compounds the target values in focus. Nevertheless, for many problems, where molecular target values need to be predicted by empirical machine learning methods, the amount of data is often scarce. Hence, in a typical prediction scenario the number of compounds with known target values can be very small (say 100 or even much below), while the number of potentially relevant features (and thus also the number of model parameters), which need to be employed initially is often large (1000 or even more). Under such circumstances, overtraining would be unavoidable unless specific precautions are applied to control and reduce the number of features and thus also the number of parameters. Becoming aware of these problems, the biological research community has started to pay increasingly attention to feature selection techniques. There are various feature selection strategies, which all have their pros and contras. Recently, feature selection using Lasso (L1) regularization has gained research interests due to its simplicity and several modifications of the original L1 regularization have been developed [4,5].

In this study we apply in a two-step regularization procedure where first L1 and than L2 regularization is applied, using L1 regularization for feature selection only. With the remaining selected features, the final model achieves higher accuracy, if it is build with L2 regularization only. In spite of its simplicity, L1 regularization requires special solvers, since the derivatives of the L1 regularization term are not defined at vanishing parameter values. A number of highly optimized solvers are available that can minimize the objective function in presence of L1 regularization [6-10]. However, the provided implementations are limited to a certain programming language and loss functions complicating re-implementation and modification of the original algorithm. In this work, the simple Rprop algorithm [11] is used to approximate the optimal L1 regularized solution leading to good results. We apply the two-step method mentioned above to the prediction tasks of CoEPrA (Comparative Evaluation of Prediction Algorithms) modeling competition of 2006 [12]. These data sets are characterized by few data points (~80) with a large number of features (~5000). The data sets of CoEPrA 2006 contain octo- and nona-peptides relevant to MHC class I binding which play an important role in the immune response of mammals. The purpose of this competition was to facilitate testing and comparison of various classification and regression algorithms for biological active molecules using blind prediction. The participant groups in CoEPrA applied various methods to the data sets at each task, and the organizers evaluated all collected predictions and then announce the rank of the participants on web site. All data sets can be obtained free of charge from the CoEPrA web site [12].

## Results

The training sets have been used to build prediction models using the proposed two-step learning method. The models have then been used to predict the provided test sets. All experiments were performed on a single core of an Intel Xeon X5670 CPU running at 2.93Ghz. Typically the whole optimization of a prediction model for one of the four CoEPrA regularization tasks required only 10 minutes of CPU time. Thereby the most CPU time demanding step is the optimization of the regularization parameters λ_1,2_, involving the average over five different ten-fold cross validations for the eight and thirteen considered candidate values of λ_1 _and λ_2_, respectively. The CPU time increases linearly with the number of features and the number of molecules in the training data set. Furthermore, the number of cross validation rounds may be decreased for large data sets. Hence, the proposed method is also applicable for much larger data sets. Finally, using the optimized prediction model requires only milliseconds of CPU time per molecule to be predicted. Hence, it can be used as a high through-put method.

We compared the achieved results with the top performing participants of the CoEPrA contest. Table 1 shows the prediction results in terms of q^2 ^values, eq. (5), on the test sets for all four CoEPrA tasks together with the optimized regularization parameters *λ*_*1 *_and *λ*_*2*_, which have been selected using the cross validation technique as described in the methods section. For all four tasks, the number of features could be reduced drastically to about one hundredth of the initial number of features or even less. Surprisingly, based on the set of features selected with L1 regularization in the first training stage, a better prediction performance is obtained, if in the second training stage L2 instead of L1 regularization is used. The prediction results of the present study surpass the best performing participants of the CoEPrA contest adopting first rank for task I and second rank for task II and III (if stage 1 is considered). As one can see from the very low q^2 ^values for task III, the prediction results are not very significant. This is due to a lack of overlap between the target values of the training and the test set as discussed below.

**Table 1:** Prediction results

rank	task I	task II	taskIII	**task IV **^**a**^
first	0.677	0.735	0.237	-2.578 (0.593)
second	0.627	0.612	0.201	-2.560 (0.565)
third	0.615	0.455	0.154	-2.561 (0.472)

**stage 1**				
λ _1_	0.05	0.05	0.08	0.1
predict	0.667	0.642	0.205	-2.573 (0.548)
features^b^	50	43	56	41
**stage 2**				
λ_2_	0.1	0.01	0.3	0.2
predict	0.691	0.668	0.131	-2.574 (0.586)

^a ^Numbers in brackets are Spearman Rank Correlation Coefficients (SRCC) [29].

^b ^number of features after L1 regularization.

Prediction results of q^2 ^values, eq. (5), for all four CoEPrA regression tasks using a two-step optimization procedure. First three lines display the results of the three best predictions for the different CEoPrA tasks. Stage 1: only L1 regularization is used. All features are removed, where the corresponding parameters have absolute values smaller than 10^-8 ^after optimization, Stage 2: only L2 regularization is applied for all features remaining after stage 1. The regularization parameters λ_1 _and λ_2 _have been determined using 5 times a 10-fold cross validation procedure.

The winner of CoEPrA regression task II and IV, and the second best for task III is the group of Curt Breneman. Their method combined the provided physico-chemical features with 147 RECON (TAE/RECON electron-density derived) descriptors. Feature selection has been performed using principal component analysis. Their final models contained 34, 148 and 148 features in task II, III and IV respectively. Wit Jakuczun, who ranked first, second and third for task III, I and IV, respectively, used a Random Forest approach for feature selection and model building [13]. For task III, his prediction model used 115 relevant features. Almost all methods used by the CoEPrA participants applied an independent feature selection step before the final model building. It is worth mentioning that none of the participants of the CoEPrA contest was able to rank best for all four regression tasks. Our prediction method provides good scores using consistently the same method for all four CoEPrA regression tasks. It serves a double purpose namely a dramatic reduction of the number of used features by feature selection and a subsequent parameter optimization in a two-step learning procedure.

**Figure 1:**
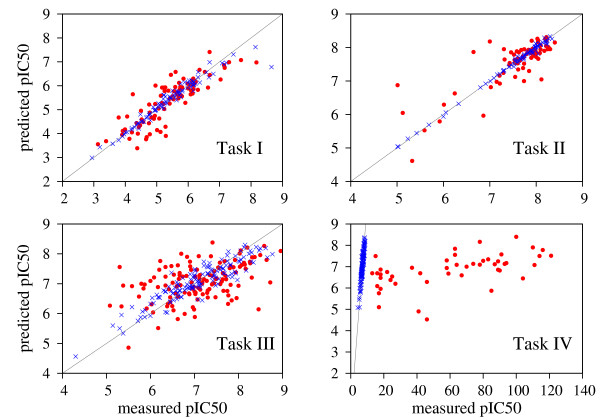
**Correlation diagrams for the four CoEPrA regression tasks**. Blue crosses: recall performance on the training set. Red dots: prediction performance on the test set.

Figure 1 shows correlation diagrams between the predicted and the measured pIC50 values for training and test sets of each CoEPrA regression task. For regression task IV the range of measured pIC50 values differs dramatically between training and test sets. Hence, it is clear that the provided training set is not a representative sample of the prediction set. Thus, the prediction performance for task IV must be very poor regardless of the methods used. For task III our approach yielded poorer results after stage 2 than after stage 1 (see Table 1). Seemingly, the initial number of features is reduced too much in this case, such that the stage 2 results are less successful. This becomes also evident comparing the regression results for the training sets, which show a large scatter for task III in contrast to the other three regression tasks. In line with this behavior, the results of all participants were relatively poor for task III indicating that this regression task is a particular difficult task.

### Feature selection via L1 regularization

**Figure 2:**
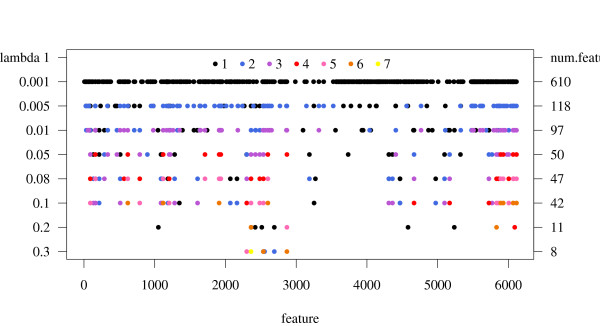
**Overview of selected features**. Feature selection with L1 regularization with increasing *λ*_1 _values (from top to bottom) for CoEPrA regression task I. Left vertical axis: *λ*_1 _Right vertical axis: number of remaining features after a cycle of L1 regularization with the corresponding *λ*_1 _value. Horizontal axis displays the feature index. The initial number of features is 6219. The colors, varying from black to yellow, exhibit how often specific features reappear in the selection procedure after they disappeared in the selection round before. For instance yellow marks features that reappeared seven times for the eight different *λ*_1 _values considered.

How the feature selection with L2 regularization works in detail is demonstrated in Figure 2. It illustrates the disappearance (by gaps) and occasional reappearance (by color code) of features with increasing λ_1 _values for CoEPrA task I. The left vertical axis represents the λ_1 _values whereas the right vertical axis shows the corresponding number of remaining features. The horizontal axis displays the 6219 initially available features. Features that have been discarded in the selection procedure correspond to white gaps. Interestingly, a very small λ_1 _value of 0.001 in L1 regularization leads already to a reduction of the number of feature by more than a factor of ten. The color code of the dots exhibits how often the corresponding features reappear with increasing λ_1 _values after they have been discarded in a previous selection round. With each addition reappearance event, the colored dot that marks the corresponding feature changes stepwise from black to yellow. Hence, dots in darker colors represent features that disappeared and reappeared rarely again whereas dots in brighter colors represent features that reappear more often. For technical reasons some dots can be covered by other dots (e.g. λ_1 _= 0.2). Therefore, the number of visible dots is smaller than the number of selected features. Interestingly, features selected at larger λ_1 _values were not necessarily selected in previous steps with lower λ_1 _values. There are no dots consistently appearing for all λ_1 _values. This demonstrates that feature selection by L1 regularization possesses some arbitrariness. We should expect such behavior for regression problems with highly redundant feature sets. Here, the composition of selected features depends strongly on the used λ_1 _value. Therefore, it is crucial to set the regularization parameter λ_1 _to an appropriate value before model building. For feature sets containing only few meaningful descriptors among a large number of meaningless features, we expect these important features to be selected for practically all λ_1 _values. This favorable property of L1 regularization suggested that it works like an oracle as discussed in some papers [14,15].

### Dependence of number of features and prediction performance

**Figure 3:**
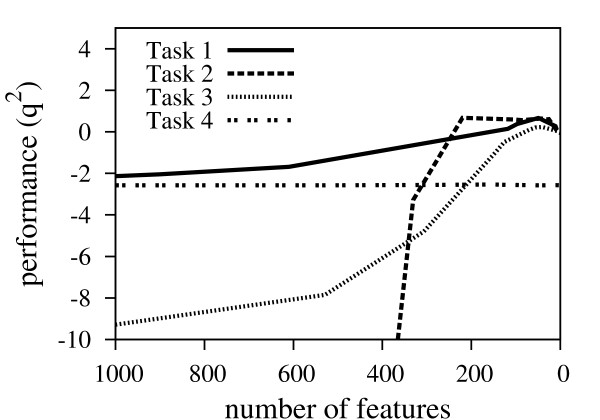
**Prediction performance during feature selection**. Prediction performance measured as q^2 ^values, eq. (5), on test set plotted versus the number of used features.

**Figure 4:**
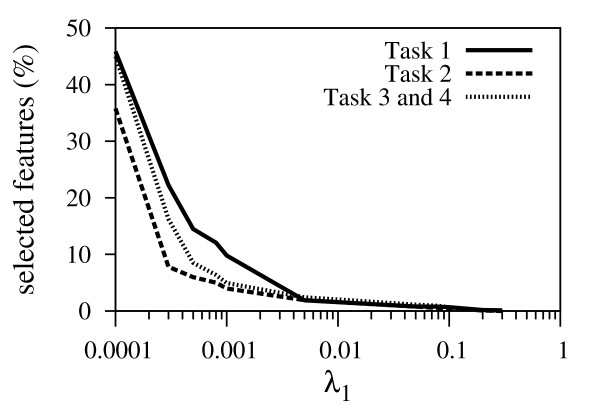
**Percentage of selected features with increasing *λ*_1 _value**. Vertical axis: percentage of selected features. Horizontal axis: selected *λ*_1 _values on a logarithmic scale.

Figure 3 shows the prediction performance for the test set measured as *q*^2 ^values versus the number of selected features for all four CoEPrA regression tasks. It is clearly seen that prediction performance increases as the number of selected features decreases up to a minimum number of features. Beyond this point, also important features are removed leading to prediction models with lower performance quality. The value of the regularization parameter λ_1 _has a strong effect on sparsity of the training data in the employed feature space. To illustrate the influence of the regularization parameter λ_1 _on the number of selected features for the CoEPrA tasks the relationship between the λ_1 _value and the ratio of selected features to the total number of features is shown in Figure 4. It can be clearly seen that with increasing λ_1 _values the number of remaining features decreases drastically. Even for small λ_1 _values only a small percentage of the initial descriptors are used.

## Discussion

In this study we applied a two-step approach using first feature selection and subsequent model building. In the first stage L1 regularization is used to filter out redundant and irrelevant features. The remaining features are used in a second stage of model building in conjunction with L2 regularization. For both steps (*p *= 1, 2) an appropriate regularization strength, governed by λ_p_, is crucial. If the regularization strength is too small, unimportant features may get a strong influence on the resulting prediction model. If it is too large, also relevant features will be removed resulting in poorer prediction performance. There are various ways to optimize the λ_p _values. In this work the λ_p _optimization has been done in an additional step using k times n-fold cross validation.

With the L1 regularization, we were able to select about one hundred or less features of the initial feature set. However, the set of selected features varied strongly with the regularization strength used to build the prediction model. We explained this behavior by the fact that these regression tasks employ feature sets with high redundancies as we used many physico-chemical features to describe each amino acid. Hence, several of these properties may be used equivalently to discriminate between amino acids. However, not only the selected number of features but also the prediction performance of the first stage depends strongly on the parameter value used for L1 regularization.

Applying L2 regularization for the selected features, we were able to achieve an even higher prediction performance for three of the four CoEPrA regression tasks. Hence, the feature selection with L1 regularization should always be followed by an L2 regularized model-building step.

Instead of a highly optimized solver to overcome the singular behavior occurring with L1 regularization, we made use of the simple Rprop algorithm [11]. Although the Rrpop method is not able to set the feature weights exactly to zero the optimal solution is well approximated. Setting a threshold near zero and removing all features with an absolute weight below this threshold seems to be a simple alternative and may also be useful for other machine learning approaches.

The choice of loss function, eq. (3), can have a large influence on prediction results. Squared error loss functions, most often used, try to recall each data point of the training set as accurate as possible. Hence, errors in the training set may have a large influence on the predictor. In this work a loss function is used which increases very smoothly with increasing prediction errors weakening the influence of potential outliers.

The data sets of the CoEPrA contest are particularly valuable, since they offer the possibility to compare the own approach with a larger number of alternative approaches from different groups on equal footing. In one case (task I) our approach surpasses the best result obtained in the CoEPrA concourse, while in two cases (task II and IV) our results are at second rank. For task III the feature selection with L1 regularization was seemingly too rigorous. Hence, the results in the second stage were of lower quality than in the first stage where the second rank was obtained. However, we like to point out the following. Although, we did not make explicit use of knowledge on the prediction set, we knew them ahead. This can have a subtle influence on the details of the procedures we were selecting in the present study and may thus have provided a hidden advantage.

Previous studies have used reduced sets of descriptors to encode amino acid sequences [16-18]. These small descriptor sets were generated using multivariate statistical methods to reduce the dimension of the feature space by Principal Component Analysis (PCA) [19]. In PCA the main assumption is that the variance of a feature represents its information content. Hence, PCA tries to project the original features into a lower dimensional space such that the variance of data in the lower dimensional space is maximized. The advantage of these generated small feature sets is that they can be used instantly to build models using few descriptors. The proposed L1/L2 method on the other hand has to be performed for each specific task (here MHC binding affinity). This results in highly optimized feature sets, which we expect to perform better than feature sets obtained with a generally valid reduction scheme. It is also possible to use PCA for a specific task similar to conventional feature selection. In this case PCA is performed on the training data of the considered problem. We used PCA analysis in this manner in previous unpublished studies. PCA was able to reduce the number of features drastically. Still, using conventional feature selection resulted in better predictive power in most cases. This may be explained by the fact that PCA combines the original descriptors such that the variance in the reduced space is maximized. However, features with large variance carry not necessarily the most useful ones for a specific learning task. Furthermore, it has to be kept in mind that PCA features in the lower dimensional space are linear combinations of the original descriptors. Hence, interpretation of selected descriptors may be much less intuitive than by the use of a classical feature selection method.

## Conclusion

The limitations of wet labs to generate larger data sets for a particular problem of interest may be due to various reasons. Mostly measurements may be expensive or complicated and time consuming to perform such as in vivo experiments. Another reason may be that the regarded problem is a quite new one. In all these cases in silico methods are highly requested as these are able to easily predict the desired target property of new compounds. Without precautions many machine learning methods fail in such situations. Hence, specialized methods are desired. Our proposed method achieved good prediction results for the four CoEPrA regression tasks. Furthermore, the number of molecular descriptors has been reduced drastically for the final prediction models. The CoEPrA data sets are representative for many biological classification and regression problems where small data sets of less than hundred are described by thousands of descriptors. Hence, we expect the proposed method to be applicable for many other machine learning tasks having same conditions.

## Methods

### Data sets

To explore model building with the two-step regularization procedure using L1 regularization followed by L2 regularization and to evaluate the performance of the proposed method, we used the data sets of the CoEPrA 2006 competition [12]. CoEPrA presented four classification and four regression tasks. For the regression tasks, binding affinities of small peptides (octo-peptides for task II and nona-peptides for all other tasks) to the class I major histocompatibility complex (MHC) have to be predicted. Each task consists of two independent data sets of oligo-peptides: a calibration data set (training set) and a prediction data set (test set). Regression task IV did not provide an independent training set. Instead the participants were asked to use the regression model from task III to make also predictions for the test set of task IV. Both data sets (for training and prediction) contain physico-chemical descriptors (explained below) and the sequences of each oligo-peptide. The calibration data sets provide also the target values, which are the measured binding affinities of the oligo-peptides to the MHC complex given as pIC50 values. The binding characteristics of the oligo-peptides making up the prediction data sets were unknown to the participants but released when the CoEPrA contest has ended. Table 2 provides an overview of all CoEPrA data sets used in this study.

**Table 2:** Overview of used data sets.

CoEPrA task	**training**^**a**^	**test**^**b**^	**L**^**c**^	**features**^**d**^
1	89	88	9	6219
2	76	76	8	5528
3	133	133	9	6219
4	133	47	9	6219

^a ^Number of ligands in training set. ^b ^Number of ligands in prediction set ^c ^Lengths of oligo-peptides for the four regression tasks.^d ^total number of considered features.

### Feature vectors

In order to build a predictor the given oligo-peptides have to be transformed into a multidimensional computer readable representation. For this purpose a fixed number of features (molecular descriptors) collected in a vector are generated for each oligo-peptide of the training and test data sets. There are several possibilities to extract feature vectors from peptide sequences. A simple method for encoding protein sequences is to assume that all 20 native amino acids have the same degree of pair-wise similarity. This so-called sparse encoding represents each amino acid of the oligo-peptide by sub-vectors of 24 components. The first 20 components are used to identify one of the 20 native amino acids. The four additional components (*,B,X,Z) are normally set to zero. However, if chemical or crystallographic analyses of the peptide or protein yield no conclusive information on the identity of the residue, one of the four additional components is set to unity. The components *,B,X,Z refer to a gap, uncertainty in asparagine/aspartate, unspecified/unknown amino acid, and uncertainty in glutamine/glutamate, respectively. For the CoEPrA data sets, this results in 216 and 192 features for nona- and octo-peptides, respectively. Since in the CoEPrA data sets all amino acids are specified, the last four components of the corresponding sub-vectors are always zero.

Ignoring chemical similarities between amino acids is a quite abrasive way to describe sequences, as it is well known that some amino acid pairs share more similarity than others do. Hence, to account for chemical similarities between amino acids BLOSUM matrices (BLOcks of Amino Acid SUbstitution Matrix) [20] have also been used to generate feature vectors. BLOSUM matrices are commonly used to score the similarity of aligned sequences. They are generated from multiple sequence alignments of protein sequences comparing the number of divergent sequences. The off-diagonal elements refer to specific amino acid pairs. As more positive a BLOSUM matrix element is, as more likely can the corresponding amino acid pair interchange in a point mutation. There are several versions of BLOSUM matrices, i.e. BLOSUM40, BLOSUM62, and BLOSUM90. The digits *XX *after BLOSUM*XX *stand for the maximum percentage sequence identity of the sequences used to generate the matrix. Hence, matrices with a large *XX *value are suitable to compare closely related sequences whereas matrices with a small *XX *value are suitable to compare distantly related sequences. The BLOSUM62 matrix yielded good performances in previous research [1] and has been used in many sequence alignment applications [21]. Therefore, in this study the BLOSUM62 matrix has been used, where every amino acid is coded using the corresponding row of the BLOSUM matrix. Analog to sparse encoding the resulting number of features for each nona- and octo-peptide using BLOSUM matrices is 216 and 192, respectively.

BLOSUM encoding describes the similarity between amino acids by a single value. A more detailed description can be achieved using physico-chemical descriptors for each amino acid type. The CoEPrA organizers provided a set of physico-chemical features for all four data sets. These features describe each amino acid by 643 physico-chemical properties taken from literature [22]. This results in a total number of 5787 or 5144 features for nona- or octo-peptides, respectively.

The different encoding techniques have their own advantages and disadvantages. Hence, a combination of the presented three encoding types (physico-chemical, sparse encoding and BLOSUM encoding) has been used in this work which resulted in 5787+216+216 = 6219 and 5144+192+192 = 5528 features for nona- or octo-peptides, respectively. Every feature encoding technique represents a different property and therefore the features are in different units. Thus, it is important to normalize the feature vectors before training a classifier. For that purpose all features with a standard deviation of zero are removed as these features do not contain any information. The remaining features are shifted and scaled such that each feature possesses a mean of zero and a standard deviation of one. The features of the test set are scaled according to the parameters derived from the training set. An overview of all used data sets together with the number of generated features is shown in Table 2.

### Linear scoring function

After feature generation, each peptide *i *of the training and predicting data set can be characterized using a corresponding feature vector ${\vec{x}}_{i}\in {\mathbb{R}}^{d}$ in a *d*-dimensional feature space. In this space a hyperplane is determined such that the distances of the training data points ${\vec{x}}_{i}$ to the hyperplane are proportional to their target values, i.e. the pIC50 values. The pIC50 values of new data points can then be predicted by computing their distances to the hyperplane. Determining the hyperplane parameters for a given data set with known target values is called supervised training. Generally, an objective function is minimized to determine the optimal hyperplane. The structure of the objective function is as follows:

(1) $$L\left(\vec{w},b\right)=\underset{model\;for\;prediction}{\underset{︸}{\frac{\left(1-\sum_{p}\lambda_{p}\right)}{N}\overset{N}{\underset{}{\sum }}\left\{\mu_{i}g\left(f\left({\vec{x}}_{i}\right),m_{i}\right)\right\}}}+\underset{regularization\;terms}{\underset{︸}{\underset{p}{\sum }\lambda_{p}{∥\vec{w}∥}_{p}}},$$

where $f\left({\vec{x}}_{i}\right)$ is a linear scoring function defined as:

(2) $$f\left({\vec{x}}_{i}\right)={\vec{w}}^{t}\cdot {\vec{x}}_{i}+b.$$

Here, $\vec{w}\in {\mathbb{R}}^{d}$ is the model parameter vector of the scoring function (hyperplane normal) and *b *∈ ℝ the threshold or bias (hyperplane offset from origin). *g*(*f*,*m*) is a so-called loss function which introduces a penalty whenever the current model does a wrong prediction on the training set depending on the error margin. In this work, we used the loss function

(3) $$g\left(f,m\right)=\text{log}\left[\text{1}+\;{\left(f-m\right)}^{2}\right].$$

This function grows very slowly with deviations of the scoring function *f *from the target value *m *and is therefore less sensitive to outliers than a quadratic loss function that is commonly used. Once a hyperplane is determined, binding affinities (pIC50 values) of oligo-peptides to a MHC receptor can be calculated using the scoring function, eq. (2).

### Regularization and feature selection

Without further knowledge, it is difficult to decide which features describe the real world objects best for a particular prediction problem. Hence, generally all features, which can be computed for the specific task, are used resulting in high dimensional feature vectors. On the other hand, the available amount of data points is quite limited especially for some biological problems. The "curse of dimensionality" states that the volume of the feature space increases rapidly as more features are added [23]. However, in many classification and regression tasks only a comparatively small number of data are available. Hence, in most cases the high dimensional feature space will be almost empty. This makes it difficult to build a model that is able to generalize for unknown data, while it may work well for the training data. Using only relevant descriptors furthermore accelerates the whole prediction process and reduces the storage space needed. Models that only depend on few features are also much easier to interpret than models with hundreds or thousands of parameters and may allow gaining more insights to the underlying chemical processes allowing chemist to modify a compound amplifying favorable properties and weakening unfavorable ones. Hence, the goal is to reduce the dimension of the feature vector space by filtering irrelevant and redundant information.

The CoEPrA competition provided such prediction problems involving many features with comparatively few data points. The participants of the CoEPrA contest used various established and new feature selection techniques such as Principal Component Analysis [24], random forests of decision trees by a genetic algorithm [25] and hybrid ant colony optimization/random forest methodology [26]. These methods vary in their complexity as well as in the number of additional algorithm specific parameters that need adjustment to obtain an optimal result. Often long-term experience and expert knowledge are necessary to set these parameters to the right values. Therefore, embedded feature selection strategies recently gained interest in the research community. In these methods feature selection and model building is combined into one single optimization step. For this purpose, the objective function contains a regularization term that constrains meaningless and redundant features to have zero weights, thus switching them effectively off. Commonly used regularization terms are related to p-norms $∥\vec{w}{∥}_{p}$ of the parameter vector $\vec{w}$, namely

(4) $$∥\vec{w}{∥}_{p}\;\;=\;\;{\left(\sum_{i=1}^{N}{\left|w_{i}\right|}^{p}\right)}^{1/p}.$$

The regularization terms used in the present study are with *p *= 1 and *p *= 2 called L1 (Lasso) and L2 (Ridge regression) regularization, respectively. Because of the quadratic form of the L2 regularization term none of the model weights will be set exactly to zero during learning and hence no explicit feature selection is done. On the other hand, Lasso regularization leads to sparse models due to its linear form where the weights of many features are set to zero rigorously, resulting in efficient feature selection.

In this work, these two regularization techniques have been used in a two-step procedure. At stage 1, the parameters are optimized using an objective function with L1 regularization. At this stage, irrelevant and redundant features are filtered out. The remaining features are used in the second stage to build a model using only L2 regularization. It is obvious that for this procedure the regularization parameters *λ*_*p *_will have a large influence on the results and are therefore chosen carefully. In this work, both parameter values are determined automatically by evaluating the prediction performance observing the error in *k *times *n*-fold cross validation. For that purpose, the training set is randomly divided into *n *parts. One part is retained as a validation set, while the other *n-1 *parts are used to train the prediction model. This process is repeated *n *times such that each part is used exactly once as validation set. The whole procedure is repeated *k *times and the average over these *k *times *n*-fold validation errors is the estimated performance. The *λ*_*p *_value that reveals the smallest validation error is then used to train the prediction model for the whole training set. In this work we set *k *= 5, *n *= 10 and the candidate sets *λ*_1 _∈ [0.001, 0.005, 0.01, 0.05, 0.08, 0.1, 0.2, 0.3] and *λ*_2 _∈ [0.0001, 0.001, 0.01, 0.1, 0.2, 0.3, 0.4, 0.5, 0.6, 0.7, 0.8, 0.9, 0.93].

### Parameter optimization

When using a least square loss function with L2 regularization the minimum of the objective function can be obtained with exact numeric solving a corresponding set of linear equations [27] with the Cholesky decomposition algorithm [28]. For all other loss functions, the parameters minimizing the objective function can only be determined approximately using for instance a gradient descent procedure.

When using the L1 regularization the objective function possesses a discontinuous derivative whenever a parameter reaches zero. Various highly optimized algorithms are capable of minimizing an objective function involving the L1 regularization term. However, the provided implementations are limited to certain programming languages and loss function types complicating re-implementation and modification of the original algorithm. In this work the simple Resilient propagation (Rprop) algorithm [11] is used to approximate the optimal L1 regularized solution. The Rprop algorithm is a quite simple though effective minimization procedure, which can be implemented in just a few lines of code. Using Rprop in conjunction with a linear regularization term does not lead to weights exactly set to zero. Nevertheless, many weights are set to nearly zero values approximating the exact solution quite well. Hence, these features can be filtered out using a low threshold. In our work, all features with an absolute weight below 10^-8 ^after the first training stage using L1 regularization have been removed before proceeding with stage 2.

### Quality assessment

Prediction performances of CoEPrA tasks I to III have been measured by the CoEPrA organizers using the coefficient of determination (q^2^) which is defined as

(5) $$q^{2}=1-\frac{{\sum_{i=1}^{n}\left(\text{pIC}50_{\exp}^{i}-\text{pIC}50_{\text{pred}}^{i}\right)}^{2}}{{\sum_{i=1}^{n}\left(\text{pIC}50_{\exp}^{i}-<\text{pIC}50_{\exp}>\right)}^{2}},$$

where $\text{pIC}50_{\exp}^{i}$ and $\text{pIC}50_{\text{pred}}^{i}$ are the measured value provided by CoEPrA and the predicted value for peptide *i*, respectively, and < pIC50exp > is the mean of all measured values. CoEPrA task IV has been evaluated using the Spearman Rank Correlation Coefficient (SRCC) [29]. However, in order to simplify comparison of prediction performances with other tasks *q*^2 ^has been computed as well for task IV.

## Authors' contributions

ODK provided the machine learning library and the initial program for the experiments and drafted the manuscript. MK carried out the experiments and drafted the manuscript. TA gave comments on the draft of the manuscript. EWK coordinated and designed the study and helped to draft the manuscript. All authors read and approved the final manuscript.
